# Around the Hospitals

**Published:** 1892-05-07

**Authors:** 


### Around the Hospitals.

The **Northallerton Cottage Hospital** has during the past year been made the headquarters and home of the nurses employed in that district by the Yorkshire branch of the Rural Nurses' Association. Eighty-seven patients received treatment in the hospital last year, contributing towards their maintenance £66, or an average of something like 3s. 8d. per week each.

**The Women and Children's Hospital, Cork,** offers an example of the increasing importance of patients' contributions towards the maintenance of such institutions. It is the most satisfactory source of contribution that exists, being a mark of the esteem in which hospitals are held, and a proof of the desire of the self-respecting poor to help themselves. There is one point in the matter which should not be overlooked, and that is, that where such efforts are made by the patients themselves, double recognition should be shown by the public by way of encouragement. Where a sum of £238 has been derived from patients' fees, surely the reliable subscriptions from the public should amount to more than £144. We hope the inhabitants of Cork will show an increasing recognition of the good services rendered by the Women and Children's Hospital, and at any rate let their subscriptions equal the patients' payments during the coming year.

More in-patients were treated at the **Ayr County Hospital** during the year ending November 22nd, 1891, than in any previous year, this being due in some measure to the increased facilities which the ambulance waggon recently acquired by the hospital has given for the removal to the hospital of patients from outlying districts. Naturally this has caused an increased expenditure—about £323 more than that of 1890, of which increase household expense s account for £253. Unfortunately, this increase in outlay has not met with a similar increase in revenue, indeed, on the contrary, the ordinary income shows a falling off of £118, so that the hospital has had to start this year's work with a debt of £441 on its maintenance account. Church collections brought in £225 to the hospital treasury, or 23 per cent. more than the sum received last year, but workmen's subscriptions showed a falling off of £14. Five hundred and fifty-three patients were admitted into the house, and 1,171 sought advice in the out-patient department.

There are two points to be noted in the report of the **New Hospital for Women,** Euston Road, for 1891. The first is that the Committee have been aroused to the importance of the annual subscriber to the well-being of the hospital, and in consequence greater efforts have been made to obtain new annual subscriptions. The result has been fairly satisfactory as this portion of the receipts shows an increase of £80 over 1890, during which year the largest amount was received that had ever been obtained from this source since the foundation of the hospital. The other point is the success of the principle of payment according to means as illustrated by this institution. All patients, and those out-patients who are not admitted by subscribers' letter are here required to make some weekly payment towards their maintenance and treatment. These payments range from the entrance fee of sixpence or a shilling and two pence a week towards medicine payable by externs, to the half-a-crown and upwards, payable by each in-patient weekly. The result of this during the past year has been that the average cost of each inmate of the hospital has been reduced by £1 4s. 5d., and out of the 2s., which it is estimated each out-patient costs the hospital, no less a sum than 1s. 10d., has been received, or, to put it in another way nearly 27½ per cent. of the total receipts has been derived from patients' payments. Altogether 304 patients were treated in the wards, and 4,436 new cases attended at the out-patient department. The expenditure during the year amounted to £2,875, but this it is expected will in future years be increased by from £400 to £500, as last year the hospital was closed for re-painting during the greater par [illegible] the summer and autumn.

